# Valedictory Address to the Graduating Class of the Baltimore College of Dental Surgery, Delivered March 4th, 1856

**Published:** 1856-04

**Authors:** Solyman Brown


### ARTICLE V.

*Valedictory Address to the Graduating Class of the Baltimore College of Dental Surgery, Delivered March 4th,* 1856. By Solyman Brown, M. D., D. D. S.

Gentlemen Graduates:

"Just twenty minutes!"—Well then, if I *ought* to confine myself to the third part of an hour, in this address to the graduating class, out of pure economy of time and patience, as I have been confidentially advised, in another city, I must at least ask the privilege of regarding the *porch* of the structure, erected merely for temporary use, as no integral part of the temple itself.

Yes—"twenty minutes and *no poetry.*" Such are the terms of the injunction kindly laid upon the speaker.

When first I received the flattering intimation that the honor of pronouncing this Valedictory Address, would be accorded to me, a dentist of the commercial metropolis, jealous peradventure of the monumental city, informed me in a friendly manner, that another member of the profession whose name was not divulged, tendered me the cautionary advice, "to limit my discourse to twenty minutes, and introduce no poetry, inasmuch as the Baltimore College is *down upon* poetry and long speeches."

This advice, whether just or not, has spared you, young gentlemen, half an hour, at least, of common time, and half a quire of "*common place.*"

So much you may pass to the credit of my unknown adviser. It is not for the speaker to decide whether you shall put the credit in the column of dollars and cents, or of mills and decimals.

The *ante-room* being thus far entered, and finding a little more opportunity to explore and expatiate, before entering the main temple of thought, allow me to admit that it is indeed a great merit, in public discourse, to condense and consolidate both thought and language.

True poetry is always such a condensation. And yet even good poetry is not necessarily suitable for all subjects and all occasions. So much we readily concede to the wisdom of our cloud-wrapt Mentor.

Acquiescing, therefore, in his charitable advice, instead of giving poetry on this occasion, in rhyming couplets, I shall read only alternate lines and alternate pages, and that will make it prose, saving you just seventy-five per cent. of the infliction which you must otherwise have sustained.

Thus far, gentlemen, be so kind as to regard yourselves as having been tantalized by the characteristic garrulity of an "old fogy," a fossilized relic of the eighteenth century, resolved on escaping by some stratagem, "the twenty minutes rule."

Lest any of you should find it difficult to imagine why a man because well stricken in years should adopt the *soubriquet* of "OLD FOGY," it ought certainly to be explained.

After searching carefully all the lexicographers, we arrive at the true etymology of this word. An OLD FOGY is one who has passed through almost all the *fogs* of life, leaving them behind him for the benefit of his juniors, himself emerging into pleasant sunshine.

And, this brings us over the threshhold, through the vestibule, into the open arena of professional speculation, where I am pleased to meet, for the first time and probably for the last, a graduating class of the Baltimore College of Dental Surgeons.

This college together with the American Journal of Dental Science, and the American Society of Dental Surgeons, constituted a few years ago, a triple confederation, with the avowed design of erecting dental surgery into an acknowledged science.

Like the Roman triumviri with Cæsar at their head, they *came* to the work, they *saw* its difficulties, and they *conquered* them.

The American society has finished its task, having been the fruitful mother of several state societies which are now successively taking her place, and performing her duties. It is doubtless best that the alma mater, venerable alike for years and usefulness, should quietly retire, and a general national convention composed of local societies, and private individuals, become her successor. Such an organization was partially initiated during the past season, with due forms and ceremonies, and it may possibly be best, that the entire profession should conspire for its success. The present is clearly an age of progress, and I doubt whether there be a solitary member of the original American Society of Dental Surgeons who would not heartily rejoice to see a better institution in its place.

What does it signify that *some* of these offspring of the superannuated parent, shall have vituperated and afflicted her, in her old age, only, that few mothers with very large families, fail to encounter the misfortune of having *some* wayward children?

"*The American Journal of Dental Science*," has nobly advanced, amidst interested obloquy and vigorous competition to its fifteenth annual volume, comprising already a complete library of professional literature, science and experience, honorable alike to its permanent editor and its constant supporters.

"*Esto perpetuo*"—may it live forever, at once the primogenitor and the contemporary of dental periodicals to the latest times. And why should it not? Can any other form or fashion of a journal, contribute more successfully to the credit, the pleasure, and the profit of the profession which it has so long and so nobly served?

Its principal contributor and conductor, through its whole course of popularity and usefulness, would have well deserved the enduring gratitude of his profession even had he never written his other unrivalled works, the "Dental Dictionary" and "Dental Practice." These two superadded to the other, constitute a *trio*, which will perpetuate his fame to very remote posterity.

Long may he live to enjoy, among other blessings, the emoluments of industry and the luxury of fame!

The first American College of Dental Surgeons, which was also the first dental college of the world, at least during the present historic era, has, in like manner, been useful, honorable and prolific. It has three living offspring, vigorous in health and honorable in renown; besides one that perished in its infancy as is sometimes the case with the *feeble* progeny of the human race.

As competition is said to be the life of trade, so mutual emulation and friendly rivalry, may, peradventure, contribute to the growth, the vigor and the permanency of all the dental colleges now in vogue. Some of them are situated very remotely from all others in the broad domain of our national confederacy, where states are like kingdoms in the old world; and consequently have ample scope, through diameters of hundreds of leagues, in a thickly populated country, for patronage and support.

The two well known facts that several dentists previously engaged in practice, have perfected their studies in these institutions, and returned to their business with redoubled popularity; and that all, or nearly all, the graduates of these colleges have been received with implicit confidence in the spheres of their practice, should induce many others of both these classes of individuals to follow their example. To a dental practitioner of either limited or unlimited preliminary advantages, and actuated by an honorable ambition, the outlay of time and money required for one or two annual courses in a dental college, would doubtless be twenty-fold repaid in future usefulness, reputation and emolument.

I am fully aware that the truth of these averments will be flatly denied by a large class of individuals who discover no personal advantage in either their acknowledgment or promulgation. But, let such persons be assured that, although they may never attain to the perspicacity of vision necessary to enable them to see, *in theory*, the truth of this doctrine, they will inevitably be implicated, *practically*, in all its issues, as surely as effects shall continue to follow causes.

If a hundred established practitioners of the younger class, and another hundred students who had never been in practice, were annually to throng the lectures and practical demonstrations of each of the dental colleges in our country, and there are none in any other land, this noble concourse would do no more than strict justice to itself, and to community, its patrons.

The practice of dentistry is destined to be elevated to higher usefulness than it has ever yet attained ; and this can be effected only by the better education of its operators.

As a class graduating at this college, and as individuals ready to enter upon the active duties of the profession, you, gentlemen, have taken, in my judgment, greatly the wiser course, by seeking and finding practical and theoretical knowledge at one of its acknowledged sources ; at the very first fountain that gushed from the chaos of the past; the veritable "pierian spring" of your professional philosophy! Used aright, this eminent advantage will bring its influence to bear upon all your future fortunes. It will give you *professional currency* wherever science is known. There is not a kingdom in the old world, nor a republic in the new, in which the degree conferred by the Baltimore College of Dental Surgeons would not impart to you all the professional authorization that either law or custom demands.

Go forth, then, where taste and duty call, accompanied by this diploma, with science in the head, energy in the heart, and skill in the hand, capable of conferring solid benefits on the community that sustains you.

Make it a point of honor and of conscience, in all your works, to give an absolute *equivalent* for the *fees* that you demand.

To do *more* is charity, which sometimes you may afford ; but to do *less* is fraud, which you never can afford: for he who shall succeed in making fraud profitable, will have countervailed all the laws of eternal providence.

This single idea opens to us the gate leading to the grand gymnasium, in which are contending all the giant and pigmy theories of dental practice. In the centre of the arena are two valiant knights, clad in massive armor, one of precious *gold*, and the other of ignoble *tin.*

Whilst Magnus Apollo favors the former by enveloping his coat of gilded mail in an atmosphere of glory, threatening to blind his adversary ; swift-winged Mercury, substituting speed for splendor, flies to the rescue of the latter, and submerges him in a *molten sea.*

In this dilemma, less the conflict should be tragic, as when Milo, of Greece, slew a Thracian bull by a single blow of his fist, and then eat him up at a meal, the valiant emperor, Caöuchouck, with his vassal kings, Rubber and Percha, interferes.

Placing these two redoubtable warriors between the gladiators, the blows are parried, the arrows repelled, the spear points broken. Thus baffled and exhausted, the combatants retire unvanquished from the field, ready for fresh encounter when they meet alone.

To drop the figure, which has become too hot for handling, we descend to simple phraseology, leaving the athlætæ and the demigods to their fabulous renown.

We find at the present day a veritable gymnasium and veritable contestants for honor and for profit in the several departments of dental practice. We find the long-established dominion of gold, employed as fillings for teeth, and as plates for the mouth, incontinently assaulted; and gutta percha, india rubber, tin, cadmium, quicksilver, "*et id omne ignobile genus*," claiming its place, and usurping its authority. And as if these were not enough to eject the most precious of these metals from this branch of art, we at length find gold itself divided against itself. We find gold in foil pitted against gold in crystals.

And what will you do, gentlemen, in this chaos of conflicting pretensions? You will doubtless listen to the universal mandate which brings all nature into order and beauty. "Let there be light."

Truth is an ocean ebbing and flowing through creation. Errors are the bubbles on its surface. The ocean remains forever inexhaustible and undiminished. The bubbles successively explode, and their gaseous elements float away from human vision.

Every science, like that which you have studied, is a vessel floating upon the sea of human knowledge; and every art, like that which you propose to practice for the benefit of mankind, yourselves included, is an aggregate of skill and experience, which manages that ship.

The bubbles of error have not all yet exploded, and you will see them on your voyage dancing on the crest of every billow. Their buoyancy will betray them. Valuable knowledge dwells far beneath the surface, as gold sinks farther in unfathomable waters, than any baser metal.

The *plain and obvious things* pertaining to dental science and practice, are readily found, and eagerly seized by idle and ignorant pretenders, and used as evidence of a *title* to professional success ; and these individuals perhaps never discover the true cause of their discomfiture. They wonder that a bubble so beautifully irridescent with prismatic tints of professional expectation, should burst and vanish in their grasp, leaving them to poverty and neglect.

The circumstance that you, gentlemen, are engaged in search of the deeper *strata* of knowledge and skill, in one of the earliest and best dental schools in our land, will clearly exonerate you from the stigma as well as the discomfiture of flippant empyricism. But as one of the probable results of your liberal course of study in a dental college, will be to render you spontaneously *conservative* of old theory and time honored practice, it may be well for you to remember that *progress* is the watchword of your camp, and *excelsior* the motto of your banner. Conservation of that which is old, should not always degenerate into rejection of all that is new. It has been well said, that the wise man acts from *reason*, the less wise from *experience* and the least wise from *authority.*

As rational men, therefore, you will be mainly governed by positive science and pure intellection; but you will not necessarily abandon reason by acting from experience; neither will you become fools by resorting to legitimate sources of information.

On bidding adieu to the faculty of the college and to each other, you will enter upon your professional duties wherever a disposing providence shall place you, as educated and authorized, and practiced operators, in the several departments of your profession. You will be confident that you have explored the stream of professional knowledge to its source; descended into its mines to their lowest stratum, and, that what you have not had an opportunity of learning in the science and art of dentistry, in its present state of advancement in our country, is not worth being either sought or known. This confidence will give you indomitable *energy*, and this energy will give you unquestionable success.

But what is success? It is easy to define what it is not.

It is not necessarily nor generally, either worldly *wealth* or contemporary *fame*, which many seek so madly. If it were, the lives of many of the Cæsars and Napoleons of the world would not have been *failures*. For although the greatest of the Cæsars was compelled to borrow 40,000 sesterces, on his departure for the war in Spain, to place his private affairs in such a state, to use his own language, "as to make him worth just nothing at all;" that is, *pay his debts:* and although the first of the Napoleons, when departing for Italy, was unable to obtain credit of his *bottier* for a pair of military boots; yet both of these personages had unbounded contemporary fame, and controlled, at times, the riches of a continent.

All desirable *success* is the attainment of *useful ends* by *honest means.* And this most blessed boon, denied forever to self-aggrandizing and unscrupulous ambition, may be easily attained by every member of the class before me, with unerring certainty.

This desirable success *may* be indeed accompanied with both fame and fortune, as was the case with Washington; although the latter is rarely acquired by the mere labor of the hands, without adventurous speculations, either in "fancy stocks," or matrimonial alliances, both of which, as avenues merely to monetary riches will be avoided by the truly wise.

*Moderate gains*, attained by patient industry and prudent economy, are all the worldly wealth of a pecuniary nature which dental practice promises to its most faithful devotees; and a settled conviction of this fact is indispensably necessary to their success. Nor is this to be regarded as matter of regret.

Enormous fortunes, like prodigious dinners, are generally burthens to their possessors. The *plethora* of either is not to be desired.

If four hundred dollars is considerably more than the portion of each individual in the common property of our country, supposing it apportioned alike to all, surely three or four thousand dollars per annum, is nothing less than *ample compensation* for the annual industry of any individual, inasmuch as this amount would absorb a portion of the general wealth equal to that of eight or ten of the non-producers, who are generally children, the aged and the infirm, the sick and the decrepid, to say nothing of the female half of the race, whose estimated earnings are classed in the columns of *duodecimals.*

Let moderation then, in all things, be your constant *safe-guard* against the fatal reaction of blasted expectations. *Hope* being the last thing that dies in man, becomes his greatest oppressor when its cold remains lie lifeless on his heart. Hope, therefore, *sanely*, and be always contented with reasonable anticipations, and normal results.

But above all things else, learn, if not known already, that there are many things in life—even in mortal life—more valuable than fame or money, the attainment of which is man's *pre-eminent success.*

What will you say of a charming conjugal companion, the breath of whose love is sweeter than the gentle south wind whispering to a bed of violets?

What will you say of a group of laughing children, clambering upon your knees, hanging upon your lips, clustering about your neck, until you imagine the riches of the universe to be gathered at your fireside?

What will you say of the esteem of the wise and good, who covet your society, and bring you in return the society of those you love?

What will you say of the consciousness of performing your daily duties with promptitude and pleasure; of doing good to others without expectation of return; of cultivating the head and heart in the knowledge of nature and the virtues of christianity; and of coming at last to the ladder of the patriarch resting on the earth, and bearing your footsteps, in company with angels, to the highest heavens!

If there be not in such blessings the elements and embodiments of success, let fame and riches be cast upon the winds; for they are useless to man.

And now, gentlemen, having obeyed the twenty minute rule, I am sure you will allow me to superimpose upon the subject, a triple crown of scientific interrogations, in the form of problems,

*to be* solved by us or our posterity. Peradventure their solution will be postponed for the exercise of the mental acumen, of the twentieth century.

The first is this. Why does the melted wax of the common honey bee, when allowed to cool slowly in a thin stratum, assume geometrical forms of the same average size and shape of the alveoli of the comb from which the wax is derived?

Is it, that the wax undergoes incipient crystallization analogous to that of the basaltic columns of Fingal's cave, and the Giant's Causeway; or, has it some undiscoverable connection with the original cells of the honey comb, and the instinct of the bee that forms them?

The second problem is this. Why do all the colors of the solar bow, or prismatic spectrum, which is simply an analysis of the sunbeam, from black to white, namely red, yellow and blue, with all their shades and combinations, enter as pigments into the human hair, blue only excepted?

Is it, that nature in her characteristic economy of material, employs this color to paint the curtains of the sky, the wavelets of the sea, the scales of the fishes, the plumage of the birds, and the petals of the flowers, and can spare no more for the human body than just enough to tint the current of the veins, and give azure eyes to a portion of the race?

Let this problem be resolved by some of our numerous descendants who will be spared by the genius of the present age, the arduous necessity of discovering circumpolar ice fields, magnetic meridians, telescopic asteroids, electric telegraphs, and manslaughtering railroads.

The third and last of these knotty problems, harder to resolve than the oracle's response, may be thus expressed.

Why did the healing art, including medicine and dentistry, have, in darker ages of the world, a greater proportion of *quack* practitioners than any other art or science?

Let posterity in its generous zeal for useful discovery, possessing facilities unknown to the present age, grapple earnestly with this question, with such success as it may be able to command. It will do well if it find the true answer by the year

1956. It will probably succeed on the same day of the same year in which some stultified geometer shall accomplish the quadrature of the circle, and determine when a traveler, starting from the equator, will arrive at the north pole, by directing his course invariably midway between the north and the west.

Of one thing, however, we feel assured, in spite of all the incertitudes and infinitudes of science, that our learned successors in the fields of discovery, in whatever age or country their conclusions shall be made, will never ascribe the charlatanry of dental practice to the timely establishment and skillful management of the Baltimore College of Dental Surgeons.

